# Hydrodistillation Extraction Kinetics Regression Models for Essential Oil Yield and Composition in *Juniperus virginiana*, *J. excelsa*, and *J. sabina*

**DOI:** 10.3390/molecules24050986

**Published:** 2019-03-11

**Authors:** Ivanka B. Semerdjieva, Santosh Shiwakoti, Charles L. Cantrell, Valtcho D. Zheljazkov, Tess Astatkie, Vicki Schlegel, Tzenka Radoukova

**Affiliations:** 1 Department of Botany and Agrometeorology, Agricultural University, Mendeleev 12, 4000 Plovdiv, Bulgaria; v_semerdjieva@abv.bg; 2 Department of Crop and Soil Sciences, Washington State University, 225 Johnson Hall, Pullman, WA 99163, USA; Santosh.Shiwakoti@wsu.edu; 3 National Center for Natural Products Research, Agricultural Research Service, United States Department of Agriculture, University, University, MS 38677, USA; clcantr1@olemiss.edu; 4 Department of Crop and Soil Science, Oregon State University, 3050 SW Campus Way, 109 Crop Science Building, Corvallis, OR 97331, USA; 5 Faculty of Agriculture, Dalhousie University, PO Box 550, Truro, NS B2N 5E3, Canada; astatkie@dal.ca; 6 Department of Food Science and Technology, University of Nebraska – Lincoln, 327 Food Technology Complex, Lincoln, NE 68583, USA; vschlegel3@unl.edu; 7 Department of Botany and Methods of Biology Teaching, Faculty of Biology, University of Plovdiv “Paisii Hilendarski”, 24 Tzar Assen, 4000 Plovdiv, Bulgaria; kiprei@abv.bg

**Keywords:** savin, eastern red cedar, greek juniper, monoterpenes, limonene, safrole, elemol, α-pinene, cedrol, sabinene

## Abstract

The chemical profile and antioxidant capacity of *Juniperus virginiana*, *J. excelsa*, and *J. sabina* essential oil (EO) fractions as a function of time was the subject of this study. The hypothesis was that, capturing EO in sequential timeframes during hydrodistillation would generate fractions containing unique compositions and antioxidant capacity. In *J. virginiana*, the highest limonene (43%) was found in the 0–5 min oil fraction, with safrole (37%) being highest in the 10–20 and 20–40 min fractions, and elemol (34%) being highest in the 160–240 min fraction. In *J. excelsa*, α-pinene (34-36%) was the highest in the 0–5 min fraction and in the control (non-stop 0–240 min distillation) oil, limonene (39%) was the highest in the 0–10 min fractions and cedrol (50-53%) was the highest in the 40–240 min fractions. In *J. sabina*, sabinene (80%) was highest in the 0–3 min fraction. The highest antioxidant capacity of *J. virginiana* was demonstrated by the 5–10 min fraction; the one in *J. sabina* by the 3–10 min fraction; and, the one in *J. excelsa*, by the control. The kinetics regression models that were developed can predict EO composition of the three juniper species eluted at different timeframes. Various industries could benefit from the results from this study.

## 1. Introduction

*Juniperus* is one of the main genera of the Cupressaceae family. Some of the species, such as *J. virginiana* L. (eastern redcedar, red cedar juniper), is widely distributed indigenous species in the eastern parts of the United States and Canada, while others, such as *J. excelsa* Bieb (Greek juniper, tree juniper) and *J. sabina* L. (savin, Cossack juniper), occupy specific and rather limited environments. *J. excelsa* prefers the Mediterranean climate and it is distributed across the Eastern Mediterranean, mainly in Northeastern Greece, Southern Bulgaria, Turkey, Syria, Iran, Lebanon, and the Caucasus Mountains [1]. *J. sabina* has a very limited, fragmented distribution range in southern and northern Europe, Crimea, Caucasus, Siberia, Kazakhstan, Mongolia, and Asia Minor, which is most probably due to its low reproductive potential. 

*Juniperus* species contain a diverse metabolic profile (coumarins, flavonoids, lignans, sterols, terpenoids, etc.) [2], which is why various extracts of these products are used in several industries. Products of different types of juniper are used in fragrances, cosmetics [3]; in the food and beverage industry [4,5]; have shown promise as ingredients in pharmaceutical products [6,7,8,9,10]; against insects [11,12]; and, as antioxidants [13,14,15].

Generally, a large variation in the chemical composition of the essential oil (EO) has been found in different juniper species due to: (1) within species and intra-population variability [16]; (2) different gender affiliation, as most junipers are dioecious [17]; (3) ecological characteristics of the habitats [18]; (4) different extraction methods [7,19,20]; (5) plant parts (leaves, galbuli, wood, or any mixes of those) [10,21,22,23]; (6) time (season) of sampling [18,19,24,25]; and, (7) the type and duration of the distillation [25,26].

*Juniperus virginiana* has been the subject of phytochemical, biological, and biosystematics investigations in a number of studies [7,16,17,18,20,27,28,29]. *J. virginiana* EO has shown activity against insects and pathogens [30,31]. Ethno-pharmacological studies for *J. excelsa* have shown that the extracts of the species were used to treat tuberculosis, jaundice, colds, coughs, and bronchitis [32,33,34]; Djeridane et al. [13] and Emami et al. [14,35] have reported antiviral and antioxidant activities. Thappa et al. [36] reported that *J. excelsa* had the potential for the commercial production of cedrol, an important aroma compound. Additionally, cedrol is the first identified oviposition attractant for African malaria vectors [37,38]. The work of von Rudloff [39] and Gao et al. [12] reflected research on the phytochemical composition of *J. sabina*. 

Generally, the industrial extraction of most EOs uses large amounts of expensive solvents (*n*-hexane, alcohol) and it requires at least 50% of the energy of the whole industrial process [40]. Future research should focus on improving the traditional steam and hydrodistillation processes and in the development of Green Extraction techniques. Green Extraction, which is a part of Green Chemistry, was defined as the extraction processes that will reduce energy consumption, allow the use of alternative solvents and renewable natural products, and ensure a safe and high-quality extract/product [40]. Our study investigated the EO fractions that were eluted at different distillation timeframe using water (as solvent) and low energy (following grinding of the plant material in water). The results from this study may contribute to the time and energy saving of EO extraction from the three juniper species, and help to obtain EO with desired composition. We hypothesized that capturing the EO segments during specific timeframes will generate EO fractions with different composition and antioxidant capacity. Therefore, in this study, the oil fractions that were eluted at different hydrodistillation timeframes and non-stop controls were compared for chemical profile and antioxidant capacity among each other within a species. The EO of each species (*J. virginiana, J. excelsa,* and *J. sabina*) were separately compared.

## 2. Results

### 2.1. Essential oil (EO) Composition of J. virginiana, J. excelsa, and J. sabina

**Figure 1:**
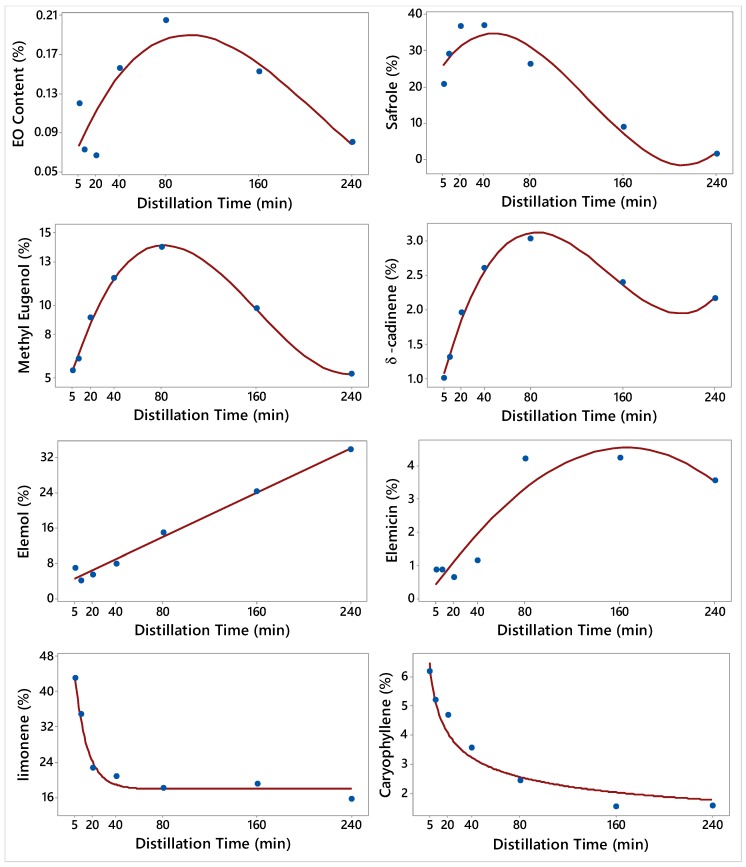
Plot of Distillation time vs. the essential oil (EO) content and the concentrations of 7 constituents along with the fitted Third order polynomial, Asymptotic, and Power regression models for *Juniperus virginiana*. The fitted models are: $\hat{Y}=0.06+0.003DT-0.00002DT^{2}+0.0000001DT^{3}$ (EO content), $\hat{Y}=23.64+0.496DT-0.0064DT^{2}+0.00002DT^{3}$ (Safrole), $\hat{Y}=4.1+0.28DT-0.002DT^{2}+0.000005DT^{3}$ (Methyl Eugenol), $\hat{Y}=0.76+0.06DT-0.0005DT^{2}+0.000001DT^{3}$ (δ-cadinene), $\hat{Y}=3.99+0.125DT$ (Elemol), $\hat{Y}=0.2+0.05DT-0.00012DT^{2}+0.0000001DT^{3}$ (Elemicin), $\hat{Y}=17.9+40.8\left(Exp\left(-0.093DT\right)\right)$ (limonene), and $\hat{Y}=11.14DT^{-0.336}$ (Caryophyllene).

The relationship between the EO content of *J. virginiana* and the concentrations of the constituents of EO with the different distillation times (DT) are illustrated in Figure 1. This figure also shows the fitted model that describes the relationship and that can be used for prediction purposes. The control treatment (non-stop 240 min distillation) resulted in 1.1% EO in dried leaves (Table 1). 

In *J. virginiana* EO, phenylpropene (Ph) was the second major chemical group, with safrole, methyl eugenol, and elemicin as major constituents (Table 1 and Table 2). Overall, the elution of EO constituents that belonged to phenylpropene increased with DT and reached highest concentrations (49.8%), in the 20-40 min fraction, and decreased afterward. The concentration of safrole (37%) was also the highest in the 20–40 min fraction, the concentration of methyl eugenol was the highest in the 40–80 min fraction (11.85%–13.97%), and the concentration of elemicin was highest in the 80–160 min fraction (4.2%) (Table 1 and Table 2).

The major chemical families of the *J. virginiana* EO constituents were monoterpene hydrocarbons (MH), phenylpropene (PH), sesquiterpenes (bicyclic, monocyclic oxygenated ST), and polycyclic aromatic hydrocarbon (PCH) (Table 3). During the first 5 min of the distillation, about 43% of the monoterpenes (e.g. limonene) were eluted. The quantity of the monoterpenes elution gradually decreased as the distillation time increased, and it reached the bottom (15.6%) at 240 min. Limonene concentration at the 0–5 min timeframe was 177% higher than that eluted at 160–240 min timeframe fraction.

Sesquiterpenes (ST, monocyclic oxygenated, bicyclic) were the third chemical group of *J. virginiana* EO constituents (Table 3). The elution of EO constituents that belonged to sesquiterpenes increased with the DT and it was the highest (35.4%) in the 160–240 min oil fraction. The concentration of elemol (33.8%) was the highest in this 160–240 min oil fraction (Table 2). The elution of caryophyllene was the greatest in the initial 5 min oil fraction and diminished in the subsequent DT fractions (Table 2). Polycyclic aromatic hydrocarbon (PCH) (δ-cadinene) was identified in small quantities; its concentration increased from 1.01% in the 5 min fraction to 3.03% in 40–80 min fraction and then dropped again to 2.16% in the 160–240 min fraction (Table 3).

Surely, the *J. virginiana* oil profile in this study was different from the constituents that were found in the *J. virginiana* wood oil reported in the literature. α-cedrene, β-cedrene, thujopsene, cuparene, cedrol, and widdrol were the major constituents of wood oil of *J. virginiana* [41]. *J. virginiana* wood oil (also known as cedarwood oil) is used in a number of consumer products, due to its unique aroma and toxicity that repel and kill many pests [42]. The concentrations of limonene and caryophyllene were high at early DT and then gradually decreased in subsequent DT (Table 1 and Table 2, Figure 1). 

**Figure 2:**
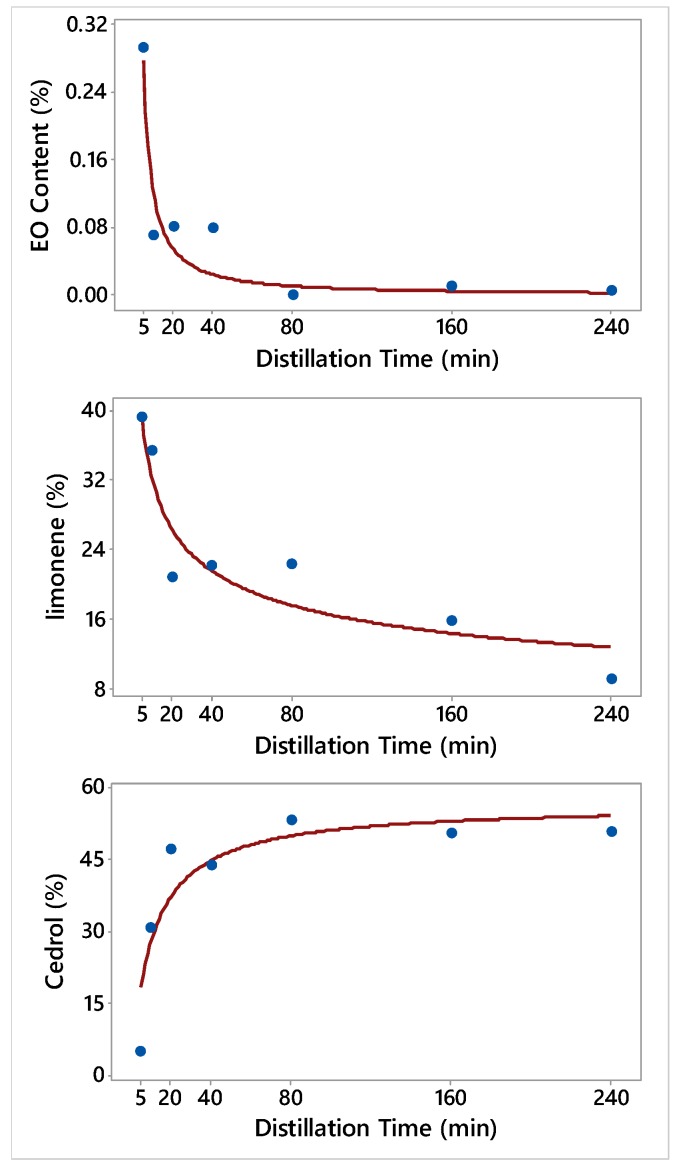
Plot of Distillation time vs. the essential oil (EO) content and the concentrations of 2 constituents along with the fitted Power and Michaelis-Menten nonlinear regression models for *Juniperus excelsa*. The fitted models are: $\hat{Y}=1.85DT^{-1.175}$ (EO content), $\hat{Y}=63.4DT^{-0.293}$ (limonene), and $\hat{Y}=\frac{56.42DT}{10.48+DT}$ (Cedrol).

Figure 2 shows the relationship of the EO fractions of *J. excelsa* and the concentrations of its constituents with the distillation timeframes (DT). *J. excelsa* EO content (yield) was significantly higher during the first 5 min (0.29%) than in the DT onward 160–240 min (Table 4). Generally, monoterpenes (α-pinene, limonene) were the predominant group of the EO constituents (Table 4 and Table 5). 

Monoterpenes were eluted early in the distillation process 0–5 min (74%) (Table 5). The concentration of α-pinene was highest at the first 5 min fraction (34%) and in the control (36%), and lowest at 10-20 min elution timeframe fraction (11%); however, there was no consistent trend in EO composition changes with progressing timeframes (Table 4). Limonene eluted early in the distillation process, and therefore its concentration was higher in the fractions that were collected earlier than in the 80–160 min or in the 160–240 min fractions (Table 4, Figure 2). Limonene concentration increased by 336% when it was collected at 0–5 min timeframe when compared to the collection at the 160–240 min timeframe (Table 4). Oxygenated sesquiterpenes (OS) were the second most predominant chemical family of the *J. excelsa* EO constituents, with cedrol acting as the main one in this group. Cedrol started eluting after 5 min in the distillation process, and therefore its concentration was the lowest in the 0–5 min fraction (4.9%). The concentration of cedrol increased by at least six-folds from 5–10 min DT (30.6%) onward when compared to 0–5 min DT (4.9%). The concentration of cedrol (53%) was the highest in the 40–80 min oil fraction (Table 4). 

**Figure 3:**
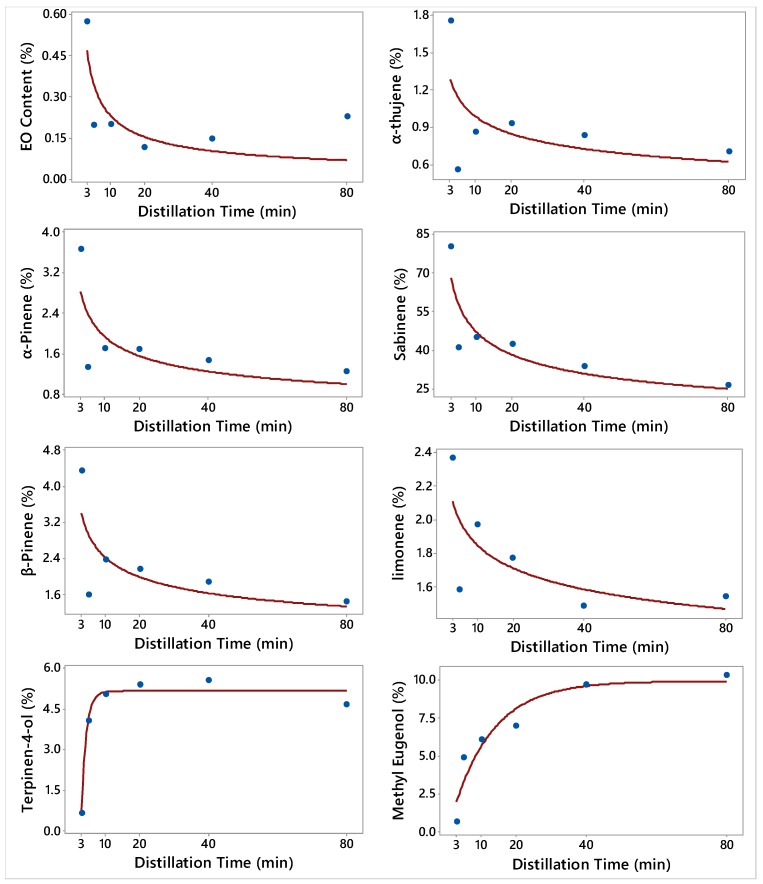
Plot of Distillation time vs. the essential oil (EO) content and the concentrations of 7 constituents along with the fitted Power (convex) and Asymptotic (convex) nonlinear regression models for *Juniperus sabina*. The fitted models are: $\hat{Y}=0.89DT^{-0.59}$ (EO content), $\hat{Y}=1.64DT^{-0.22}$ (α-thujene), $\hat{Y}=4.0DT^{-0.31}$ (α-pinene), $\hat{Y}=94.8DT^{-0.3}$ (Sabinene), $\hat{Y}=4.67DT^{-0.28}$ (β-pinene), $\hat{Y}=2.38DT^{-0.11}$ (limonene), $\hat{Y}=5.16-36.3\left(Exp\left(-0.696DT\right)\right)$ (Terpinen-4-ol), and $\hat{Y}=9.94-10.3\left(Exp\left(-0.087DT\right)\right)$ (Methyl Eugenol).

The relationships between the EO of *J. sabina* and its constituents, and DT, are illustrated in Figure 3. The fitted Power (convex) and Asymptotic models that describe these relationships are also shown in Figure 3. The distillation timeframes for capturing the oil fractions for *J. sabina* were selected to be different from those of *J. virginiana* and *J. excelsa* in this study based on preliminary studies. The main chemical group of *J. sabina* EO was the monoterpenes (hydrocarbons and оxygenated) (Table 6 and Table 7). A significant portion of the monoterpene hydrocarbons (MH, 90.5%) in *J. sabina* was eluted very early in the distillation process that constituted the 0–3 min fraction (Table 8). As the name suggests, sabinene was the major constituent of the MH in *J. sabina* EO (Table 6). Sabinene was eluted early in the distillation process (Figure 3). Therefore, the concentration of sabinine was greater in the 0–3 min fraction (80%) and it decreased in the fractions that were collected later in the distillation process (26.6%) (Table 6). Similarly, the 0–3 min fraction had higher concentrations of α-pinene, β-pinene, α-thujene, and limonene when compared to fractions that were collected at the late elution timeframes (after 3 min) (Figure 3, Table 6 and Table 7). 

The concentration of citronellic acid was the lowest during the first 3 min elution (2.4%), peaked during the 3–5 min (11%), and then decreased with subsequent DT.

Methyl eugenol was the major constituent of the third chemical group, phenylpropanoids of *J. sabina* EO. The concentration of methyl eugenol was the lowest in the 0–3 min fraction (Figure 3, Table 7) and it increased in the subsequent timeframes. Terpinen-4-ol was the major constituent of the oxygenated monoterpenes (OS) chemical group, and its concentrations increased to around 5.5% in the 20–40 min fraction.

### 2.2. Antioxidant Capacity of J. virginiana, J. excelsa, and J. sabina

The in vitro anti-oxidant capacity of *J. virginiana*, *J. excelsa*, and *J. sabina* was found to be affected by the DT (Table 9), thus confirming our hypothesis. The oxygen radical absorbance capacity (ORAC) value of *J. virginiana* was the highest at the 5–10 min time fraction (329 μM Trolox Equiv/per g oil) and the lowest at the 40–160 min timeframe (184 μM Trolox Equiv/per g oil) (Table 9). Similarly, the ORAC value of *J. sabina* was the highest in the 3–10 min oil fraction (56 μM Trolox Equiv/per g oil) and the lowest at the 10–20 min oil fraction (22 μM Trolox Equiv/per g oil). The significant trend with DT was observed for the ORAC value of *J. excelsa,* indicating that the in vitro antioxidant capacity of *J. excelsa* drastically decreases with subsequent elution (Table 9). However, greater anti-oxidant capacity from the EO of *J. excelsa* can be captured if the EO is collected in 240 min, i.e., 0–240 min non-stop DT (104 μM Trolox Equiv/per g oil) (Table 9).

## 3. Discussion

This is the first report on *J. virginiana*, *J. excelsa*, and *J. sabina* EO fractions that were generated in different timeframes following a grinding of the material to significantly speed up the extraction. Overall, the results confirmed the hypothesis of this study that capturing the EO segments during specific timeframes will generate EO fractions with different composition and antioxidant capacity (ORAC values).

### 3.1. Essential Oil (EO) Composition of J. virginiana, J. excelsa, and J. sabina

The EO yield (content) of the leaves of *J. virginiana* at different DT frames that were used in this study was higher than the *J. virginiana* EO yield that was reported by Gawde et al. [28,29], who recovered 0.17% of EO after 90 min of steam distillation, whereas *J. virginiana* EO yield in this study was 1.12% (in the control). Therefore, this study revealed that the grinding of *J. virginiana* leaves in water prior to extraction with subsequent hydrodistillation for 240 min can recover > six fold of EO as compared to the 90 min steam distillation without grinding, as in the study by Gawde et al. [28]. In the study of Cantrell et al. [16], the *J. virginiana* ‘Grey Owl’ and *J. virginiana* ‘Canaertii’ the EO yield was 0.5–0.65% and 0.04–0.3%, respectively, demonstrating the range of EO yield in this species.

The specific composition of the EO fractions that were eluted at different hydrodistillation timeframes can be attributed to the different boiling points of the respective EO constituents [43,44]. The EOs are complex products and they include a number of constituents with different molecular weights and diverse temperature separation points. In the initial minutes of the distillation, the most volatile EO constituents, such as monoterpenes, are eluted. Limonene (monoterpene) and caryophyllene (sesquiterpenes) eluted at early DT due to their low boiling points (limonene at 176 °C and caryophyllene at 116 °C), whereas elemol increased in late timeframe fractions due to its high boiling point (290 °C). 

The concentration of limonene that was collected at the first 5 min in this study was 2.4 and 16.6 times greater than that reported by Dunford et al. [45] and Gawde et al. [29] from Oklahoma and Mississippi, USA, respectively. The former authors had used hydrodistillation for 6 h, while the latter researchers had used steam distillation for 1.5 h. In addition to the extraction methods implied, the ecotype of plant affects the composition of EO [46]. However, it is evident from this study that the highest concentrations of the major constituents of *J. virginiana* leaves (limonene and safrole) can be collected with minimal time and energy with this method of prior grinding of the material in water. Limonene, safrole, and elemol were the major constituents of *J. virginiana* in this study, and the overall oil profile was similar to some of the *J. virginiana* oils in the samples that were collected from 49 locations in the United States [16]. However, the *J. virginiana* oil in this study would not fit any of the 10 chemotypes that were identified by the latter authors. Therefore, we could classify *J. virginiana* that was used in this study as another chemotype of *J. virginana*, namely limonene-safrole-elemol chemotype. 

In this study, the major chemical families of *J. excelsa* control oil included monoterpene hydrocarbons (MH, such as 35.6% α-pinene and 30.6% limonene) and oxygenated sesquiterpenes (OS, 33.8% cedrol). The concentration of α-pinene in this study was within the range for *J. excelsa* EO that was reported by Sanjani et al. [24], however, the concentration of limonene in the same report was only 1.5–2.1%, and no cedrol was reported, which is difficult to explain. Unlu et al. [22], reported α-pinene (55.5%), α-cedrol (7.7%), sabinene (3.5%), and verbenone (2.4%) as the main constituents of *J. excelsa* galbuli EO.

Also in this study, higher concentrations of the major constituents of the *J. excelsa* EO eluted in significantly shorter distillation times than in the study by Emami et al. [14]. It took four hours of distillation for Emami et al. [14] to collect *J. excelsa* oil with 32% α-pinene, 4.4% limonene, and 13% cedrol when compared to this study, where most of the oil was eluted in the first 5 min. In this study, cedrol in the 5–10 min fraction was already 31%. Similarly, sabinine, a major constituent of the EO of *J. sabina*, also eluted at a significantly higher concentration in the first 3 min than in the subsequent DT. Differences may be attributed to grinding of the plant material prior to the EO extraction in this study. The results from this study can be used to specify the optimum distillation time and to avoid the unnecessarily long DT (if the material is ground prior to the EO extraction). *J. excelsa* EO with high concentrations of α-pinene and limonene and low concentration of cedrol can be obtained in the first 5 min DT. Conversely, if greater than 50% cedrol is desirable, then this can be achieved by removing the first 5 min eluted fraction. Cedrol is a well-known and widely used aromatic ingredient in various consumer products, as in perfumery and cosmetics, shampoos, soaps, cleaners and detergents [47], due to its mild woody cedarwood-like sweet aroma with a sandalwood note. In addition, cedrol has shown a preventive effect against chemo-therapy induced alopecia in mice [48] and it has shown insecticidal properties [31,47], it has a potential to be used for preventing or treating autoimmune diseases [49]. Worldwide consumption/demand for cedrol has been reported to be 1–10 metric tonnes per year [50].

In this study, sabinene was the main *J. sabina* EO constituent and it belongs to the monoterpenes group. The control *J. sabina* oil had 61% sabinene. This study demonstrated that a high sabinene EO fraction (80% sabinene) could be obtained in the first 0–3 min hydrodistillation time, following a grinding of plant material in water. The concentration of sabinene in this study was higher than the values reported in the literature, whereas the concentration of the other monoterpene constituents, such as α-thujene, α-pinene, β-pinene, limonene, terpinen-4-ol, and the phenylpropanoids citronellic acid ME and methyl eugenol, were generally lower. 

In a study with male and female plants *J. sabina* leaves, Asili et al. [51] reported sabinene (22 and 24%), α-pinene (15 and 6%), and myrcene (7 and 8%), respectively. Fournier et al. [52], in a study of *J. sabina* cultivars and wild type, reported 24% sabinene in the wild type, and 18%, 18%, and 41% in three *J. sabina* cultivars. In the same study, sabinyl acetate was 45.5% in the EO of the wild type, and 53%, 19%, and 38% in the three *J. sabina* cultivars, respectively. The authors commented that sabinene in previous studies varied from 26 to 42% of the oil. However, the data that was provided seemed to be from a single rep and it was lacking statistical analyses. Apparently, the *J. sabina* chemical composition and the concentration of sabinene widely vary, which suggests the presence of chemotypes. We anticipate more research on *J. sabina* accessions and the identification of various chemotypes. From practical perspective, high sabinene (80%) low methyl eugenol *J. sabina* EO fraction can be obtained if the oil is sampled in the first 3 min of the distillation process. In a recent ruling, the United States Food and Drug Administration (FDA) [53] removed six synthetic flavoring substances, including methyl eugenol, from the food additives list, because experiments with high intake doses may cause cancer in laboratory animals. Although this ruling did not affect “natural flavors” counterparts that were extracted from plants, we expect the industry to diminish/reduce the use of methyl eugenol and/or reduce its concentration in food products. Therefore, we anticipate increased market demand for EO with reduced methyl eugenol content.

### 3.2. Antioxidant Capacity of J. virginiana, J. excelsa, and J. sabina Essential Oils (EO)

Essential oils have been reported to possess anticancer, antinociceptive, antiphlogistic, antiviral, antibacterial, and antioxidant properties [54]. Studies that seek to reveal the antioxidant potential of natural products, including EO, are numerous, although the in vitro antioxidant activity assays have a number of drawbacks and do not allow for a comparison of the results [55,56]. Furthermore, it is now clear that the term ‘antioxidant’ is primarily a marketing tool. The results from in vitro antioxidant capacity assays have little if any relevance to the complexity of the interactions of EO in biological systems. The higher antioxidant capacity of some natural products that were observed in vitro cannot be readily correlated to a potential positive health effect on humans, leading some scientists to suggest the banning of these types of assays [56], and some scientific journals to declare that they would no longer review manuscripts describing in vitro antioxidant measurements [55]. 

Generally, the in vitro antioxidant potential of the EO depends on its composition and it is determined by the interaction between its constituents [57]. It is known that phenols and secondary metabolites with conjugated double bonds usually exhibit significant in vitro antioxidant activity [58]. Secondary metabolites with conjugated double bonds also include monoterpenes and sesquiterpenes in the EO. However, it is impossible to model the specific contribution of each EO constituent towards the overall in vitro antioxidant capacity of a given fraction. Further experiments would be needed to assess the observed in vitro antioxidant capacity of individual EO constituents, because, in many cases, there is synergy between the various EO constituents with respect to their bioactivity [57,59]. 

Overall, in this study, the higher in vitro antioxidant capacity was exhibited by the 3–10 min oil fraction in *J. sabina* and by the 5–10 min oil fraction in *J. virginiana*. In *J. sabina*, monoterpenes (α-pinene, β-pinene, limonene) and sesquiterpenes (α-thujene, sabinene) predominated in this fraction. In *J. virginiana*, limonene and caryophyllene phenylpropene (safrole), branched unsaturated hydrocarbons (Pregeijerene), predominated in this time fraction. Cantrell et al. [16] reported a difference in *in vitro* antioxidant activity on EO of *J. virginiana,* due to differences in chemotypes. The antioxidant capacity of the EO may also change with the change in the ratio of various volatile and non-volatile compounds [60]. Zheljazkov et al. [61] found the relation of composition of EO with the in vitro antioxidant capacity of *J. sabina* and *J. excelsa*; and, Emami et al. [14] also observed variation in the antioxidant capacity of the EO of *J. excelsa* with the methods of the antioxidant assays employed. Our study revealed that in vitro antioxidant capacity depends on the specific composition of the oil fraction that was captured in distinct timeframe, and hence support our hypothesis. However, the results from the in vitro antioxidant capacity of EO fractions may only be indicative of differences in some kind of activity between the fractions, and it must not be correlated to any potential health effects of a specific EO fraction. 

## 4. Materials and Methods

### 4.1. Plant Material

Plant material of *Juniperus excelsa* Bieb., *Juniperus sabina* L., and *Juniperus virginiana* L. (branches not thicker than 10 mm with leaves) was collected in the autumn of 2017 from natural populations (of the *J. sabina* and *J. excelsa*) in Bulgaria. The collected biomass samples were immediately transferred and then dried in an aerated shady place for a month until a constant weight, before oil was isolated. The leaves were carefully separated from branches in order to avoid EO losses. Therefore, in this study, only the leaves of the three junipers were distilled for EO extraction. The collection of juniper biomass samples was made from the following habitats: *J. excelsa* biomass samples were collected from the natural habitat near the town of Kresna, Bulgaria, along the road at 41°046′00.1″N; 23°08′55.5″E. The *J. virginiana* biomass samples were collected along the road from the town of Blagoevgrad to Simitli, Bulgaria, collected at 41° 51′17.57″N, 23° 07′48.72″E. It was assumed that the specific *J. virginiana* tree was either an escapee or planted as ornamental in that area. The *J. sabina* biomass samples were collected from natural habitat near Beli Iskar, Bulgaria, at 42°15′46″.3″N, 23° 32′26″.7″E. The voucher specimens of *J. excelsa* Bieb., *J. sabina* L., and *J. virginiana* L. (small branches with needles) were deposited at the Herbarium of the Agricultural University, Plovdiv, Bulgaria (SOA) [62].

### 4.2. Essential Oil (EO) Extraction of the Juniper Leaves

The EO of the leaves was extracted via hydrodistillation in 2-L distillation units (Laborbio Ltd. Sofia, Bulgaria, laborbio.com) at the Research Institute for Roses and Medicinal Plants in Kazanluk, Bulgaria. Each extraction was performed in three replicates. Research has shown that the juniper EO may continue to elute even after 10–14 h of steam distillation [18,25]. Therefore, to speed up the hydrodistillation process, the samples were ground in water prior to the extraction. Samples of 100 g of dried leaves plus 1.2 L of water were placed in a kitchen food processor (blender) and ground for 48 s immediately prior to the extraction. Our preliminary studies demonstrated that grinding juniper leaves in water greatly reduces the time and energy necessary for the EO extraction, and also eliminates EO losses due to the potential rapid volatilization during the grinding process. 

The beginning of the distillation in each replicate was noted when the first droplet of EO dropped from the condenser into the collecting unit of the apparatus. The EO fractions were captured at different timeframes: 0–5; 5–10; 10–20; 20–40; 40–80; 80–160; 160–240; and, 0–240 non-stop control for *J. virginiana* and *J. excelsa* and 0–3; 3–5; 5–10; 10–20; 20–40; 40–80; and, 0–80 min non-stop control for *J. sabina*. These timeframes were established based on preliminary experiments. The eluted oil fractions were captured without interrupting the hydrodistillation process, resulting in EO fractions that represented the eluted oil constituents within these timeframes. The oil was transferred in 2-mL vials and placed in a freezer. Later, the oil was separated from water and measured on an analytical scale and kept in a freezer again until the oil was analyzed. Here, we report the oil content (yield) based on weight. 

### 4.3. Gas Chromatography Mass Spectrometry Flame Ionization Detection (GC-MS-FID) of essential oil (EO) and Distillation Fractions

The constituents were identified and quantified in juniper essential oil and distillation fractions. Oil samples were analyzed by GC-MS-FID on an Agilent 7890A GC system (Santa Clara, CA, USA) that was equipped with a Agilent 5975C inert XL MSD with triple axis detector and an Agilent 7693 autosampler. DB-5 fused silica capillary column (30 m × 0.25 mm, with a film thickness of 0.25 μm) was used and operated using the following conditions: injector temperature, 240 °C; column temperature, 60–240 at 3 °C/min, and then held at 240 °C for 5 min; carrier gas, He; injection volume, 1 μL (split ratio 25:1); the FID temperature was 300 °C. Post-column splitting was performed so that 50% of sample proceeds to FID and 50% to mass spectrometry (MS) detection. The MS mass range was from *m*/*z* 50 to 550; filament delay, 3.5 min; source temperature, 230 °C; and, quad temperature, 150 °C. 

Kovat analysis identified the compounds limonene, safrole, methyl eugenol, caryophyllene, α-pinene, cedrol, sabinene, β-pinene, terpinen-4-ol, and methyl eugenol in oil samples [63], comparison of retention times and mass spectra with authentic standards, and a comparison of mass spectra with those that were reported in the NIST mass spectra database. Standards of R-(+)-limonene, safrole, methyl eugenol, caryophyllene, α-pinene, cedrol, sabinene, β-pinene, terpinen-4-ol, and methyl eugenol were purchased from Sigma–Aldrich (St. Louis, MO, USA). Compounds pregeijerene B, α-thujene, δ-cadinene, elemol, and elemicin were identified in oil samples by Kovat analysis and a comparison of mass spectra with those that were reported in the NIST mass spectra database and/or comparison of mass spectra with those reported by Adams et. al., 2007 [63]. Citronellic acid methyl ester was identified by a comparison of mass spectra with those that were reported in the NIST mass spectra database and by a comparison of retention time and mass spectra data with an authentic standard that was synthesized in our laboratory. Citronellic acid methyl ester was produced by methylation of (S)-(−)-citronellic acid (Sigma–Aldrich (St. Louis, MO, USA) using diazomethane. 

Compounds were quantified by performing area percentage calculations based on the total combined FID area. For example, the area for each reported peak was divided by the total integrated area from the FID chromatogram from all reported peaks and multiplied by 100 to arrive at a percentage. The percentage of a peak is a percentage relative to all other constituents that were integrated in the FID chromatogram. 

### 4.4. Diazomethane Generation and Citronellic Acid Methyl Ester Synthesis

An Aldrich Mini Diazald apparatus was used for the production of CH_2_N_2_ in ether. Briefly, 2.5 g of KOH was dissolved in 4 mL of deionized H_2_O and then placed in the reaction vessel, followed by the addition of 5 mL of EtOH. A separatory funnel containing 2.5 g of diazald dissolved in 22.5 mL of ether was placed above the reaction vessel. The reaction vessel was warmed to 68 °C using a H_2_O bath, followed by the drop wise addition of the diazald soln. over a period of 40 min. The receiving flask and condenser cold finger were cooled using a dry ice/acetone bath. The co-distilled CH_2_N_2_ in ether soln. was stored in sealed vials at −20 °C until needed. 

2.5 mg of (S)-(−)-citronellic acid in 0.5 mL of methylene chloride was treated at r.t. with a soln. of 0.5 mL of CH_2_N_2_ in the ether prepared above. The soln. was placed in a laboratory fume hood overnight to complete the reaction, allowed for evaporation of solvent and CH_2_N_2_, and the sample was then redissolved in Et_2_O for GC analysis.

### 4.5. Methodology for Antioxidant Capacity Evaluation of the Essential Oils (EO) Fractions from the Three Juniper Species 

The EO fractions from the three junipers were analyzed for antioxidant capacity using the oxygen radical absorbance capacity (ORAC oil) at the University of Nebraska-Lincoln, Small Molecule Analysis Laboratory, using the method that was developed by Huang et al. [64,65] and as described previously [61]. Briefly, Trolox, (6-hydroxy-2,5,7,8-tetramethylchroman-2-carboxylic acid), which is a polar derivative of Vitamin E, was used as a standard, and the results were reported as µmole Trolox g^−1^. Each replicate of the EO from the three junipers and all DTs were analyzed in triplicate, and the averages of these three readings were used for the statistical analysis, as described below. 

### 4.6. Statistical Analyses 

The effect of distillation time (DT) on EO content and the concentration of constituents were determined for each of three juniper species (*J. virginiana*, *J. excelsa*, and *J. sabina*) in Bulgaria using a one-way analysis of variance. For *J. virginiana*, the constituents were limonene, pregeijerene B, safrole, methyl eugenol, caryophyllene, δ-cadinene, elemol, and elemicin. For *J. excelsa*, the constituents were α-pinene, limonene, and cedrol. For *J. sabina*, the constituents were α-thujene, α-pinene, sabinene, β-pinene, limonene, terpinen-4-ol, citronellic acid methyl ester, and methyl eugenol. For each species, the effect of DT on ORAC value (uM Trolox Equiv/per g oil) was also determined.

For each response variable, the validity of model assumptions was verified by examining the residuals, as described in Montgomery [66]. Since the effect of DT was significant (*p*-value < 0.05) on all response variables, multiple means comparison was completed using Tukey’s Multiple Range test at the 5% level of significance and letter groupings were generated. The analysis was completed using the GLM Procedure of SAS [67]. 

For *J. virginiana*, the most appropriate regression model that describes the relationship between DT and EO content, concentrations of safrole, methyl eugenol, δ-cadinene, elemol, and elemicin was a third order polynomial (Equation (1)), the model that describes the relationship between DT and the concentration of limonene was Asymptotic (convex) (Equation (2)), whereas the model that describes the relationship between DT and the concentrations of caryophyllene was Power (convex) (Equation (3)). There was no relationship between DT and pregeijerene B. 

For *J. excelsa*, the most appropriate regression model that describes the relationship between DT and EO content, as well as the concentration of limonene, was Power (convex) (Equation (3)), whereas the model that describes the relationship between DT and the concentration of cedrol was Michaelis–Menten (Equation (4)). There was no relationship between DT and the concentration of α-pinene. 

For *J. sabina*, the most appropriate regression model that describes the relationship between DT and EO content, as well as the concentrations of α-thujene, α-pinene, sabinene, β-pinene, and limonene, was Power (convex) (Equation (3)), whereas the relationship between DT and the concentrations of terpinen-4-ol and methyl eugenol was best described by the Asymptotic (convex) (Equation (2)) model. There was no relationship between DT and the concentration of citronellic acid methyl ester.

(1) $$Y=\beta_{0}+\beta_{1}X+\beta_{2}X^{2}+\beta_{3}X^{3}+\varepsilon$$

(2) $$Y=\theta_{1}-\theta_{2}\left(\exp(-\theta_{3}X\right))+\varepsilon$$

(3) $$Y=\theta_{1}X^{\theta_{2}}+\varepsilon$$

(4) $$Y=\frac{\theta_{1}X}{\theta_{2}+X}+\varepsilon$$

where Y is the dependent (response) variable, X is the independent (DT) variable, and the error term ε is assumed to have normal distribution with constant variance. 

While the third-order polynomial model (Equation (1)) is linear, the other three models (Asymptotic, Power, and Michaelis–Menten) are nonlinear and their parameters were estimated iteratively using the NLIN Procedure of SAS [67], and the fitted models met all the adequacy requirements of nonlinear models [68]. The figures, as well as the third-order polynomial model, fits were prepared using Minitab 18 software (Minitab, State College, PA, USA).

## 5. Conclusions

This experiment enabled to model the elution of various EO constituents and to pinpoint the oil fractions of *J. virginiana, J. excelsa,* and *J. sabina* with the specific oil composition and the EO with the highest in vitro antioxidant capacity at different distillation timeframes. The study demonstrated that, by manipulating DT and capturing fractions at specific time points, one can capture the desired composition of EO with less time and energy. The results of this study could be a significant finding for the pharmaceutical, aromatic, and other industries that use the EO of *J. virginiana, J. excelsa,* and *J. sabina*.

## Figures and Tables

**Table 1:** Mean *Juniperus virginiana* essential oil (EO) content (%), and the concentrations (%) of limonene, pregeijerene B, safrole, and methyl eugenol in the EO fractions obtained from the eight distillation times (DT).

DT (min)	EO Content (%)	Limonene (%)	Pregeijerene B (%)	Safrole (%)	Methyl Eugenol (%)
5	0.12 b ^1^	43.2 a	5.87 bc	20.60 bc	5.48 e
10	0.07 b	34.9 ab	9.30 a	29.00 ab	6.28 de
20	0.07 b	22.7 bc	8.45 a	36.50 a	9.12 c
40	0.16 b	20.7 bc	7.03 b	36.79 a	11.85 ab
80	0.20 b	18.1 bc	6.59 b	26.15 bc	13.97 a
160	0.15 b	19.1 bc	5.93 bc	8.94 d	9.75 bc
240	0.08 b	15.6 c	4.80 c	1.45 d	5.24 e
Control	1.12 a	26.9 bc	6.80 b	19.08 c	8.53 cd

^1^ Within each column, means followed by the same letter are not significantly different at the 5% level of significance.

**Table 2:** Mean *Juniperus virginiana* concentrations (%) of caryophyllene, δ-cadinene, elemol, and elemicin in the essential oil fractions obtained from the 8 distillation times (DT).

DT (min)	Caryophyllene (%)	δ-Cadinene (%)	Elemol (%)	Elemicin (%)
5	6.17 a ^1^	1.01 d	6.90 de	0.86 b
10	5.19 b	1.31 d	4.15 e	0.86 b
20	4.68 b	1.95 c	5.43 de	0.65 b
40	3.55 c	2.60 ab	7.92 d	1.16 b
80	2.42 de	3.03 a	14.90 c	4.20 a
160	1.54 f	2.39 bc	24.30 b	4.23 a
240	1.56 ef	2.16 bc	33.84 a	3.55 a
Control	2.84 cd	2.09 bc	15.05 c	3.41 a

^1^ Within each column, means followed by the same letter are not significantly different at the 5% level of significance.

**Table 3:** Chemical families of *Juniperus virginiana* essential oil (EO) constituents as a function of their elution during hydrodistillation timeframes.

DT (min)	0–5	5–10	10–20	20–40	40–80	80–160	160–240	Control 0–240
MH%	43.2	34.9	22.7	20.7	18.1	19.1	15.6	26.9
PCH%	1.01	1.31	1.95	2.60	3.03	2.39	2.16	2.09
Ph	26.94	36.14	46.27	49.8	44.32	22.91	10.24	31.02
ST	13.07	9.34	10.11	11.47	17.32	25.84	35.4	17.89

DT—Distillation time; MH—Monoterpene hydrocarbons; PCH—polycyclic aromatic hydrocarbons; Ph—phenylpropene; ST—sesquiterpenes.

**Table 4:** Mean *Juniperus excelsa* essential oil (EO) content (%), and the concentrations (%) of α-pinene, limonene, and cedrol in the EO fractions that were obtained from the eight distillation timeframes (DT).

DT (min)	EO Content (%)	α-Pinene (%)	Limonene (%)	Cedrol (%)
5	0.29 b	34.4 a	39.2 a	4.9 b
10	0.07 c ^1^	19.7 ab	35.3 ab	30.6 ab
20	0.08 c	11.1 b	20.7 abc	47.1 a
40	0.08 c	16.4 b	22.1 abc	43.8 a
80	0.00 c	24.7 ab	22.3 abc	53.1 a
160	0.01 c	24.0 ab	15.8 bc	50.3 a
240	0.01 c	13.9 b	9.0 c	50.5 a
Control	0.93 a	35.6 a	30.6 abc	33.8 ab

^1^ Within each column, means followed by the same letter are not significantly different at the 5% level of significance.

**Table 5:** Chemical families of *Juniperus excelsa* essential oil (EO) constituents as a function of their elution during hydrodistillation timeframes.

DT (min)	0–5	5–10	10–20	20–40	40–80	80–160	160–240	Control 0–240
MH%	73.6	55.0	31.8	38.5	47.0	39.8	22.9	66.2
ST	4.9	30.6	47.1	43.8	53.1	50.3	50.5	33.8

DT- Distillation time; MH - Monoterpene hydrocarbons; ST- sesquiterpenes.

**Table 6:** Mean *Juniperus sabina* essential oil (EO) content (%), and the concentrations (%) of α-thujene, α-pinene, sabinene, and β-pinene in the EO fractions obtained from the seven distillation timeframes (DT).

DT (min)	EO Content (%)	α-Thujene (%)	α-Pinene (%)	Sabinene (%)	β-Pinene (%)
3	0.57 b ^1^	1.75 a	3.67 a	80.1 a	4.36 a
5	0.19 c	0.55 b	1.33 b	41.2 bc	1.60 bc
10	0.19 c	0.85 b	1.70 b	45.1 bc	2.38 bc
20	0.11 c	0.93 b	1.69 b	42.4 bc	2.18 bc
40	0.14 c	0.82 b	1.48 b	33.7 c	1.88 bc
80	0.22 c	0.70 b	1.25 b	26.6 c	1.45 c
Control	1.43 a	1.36 ab	2.39 ab	61.4 ab	3.67 ab

^1^ Within each column, means followed by the same letter are not significantly different at the 5% level of significance.

**Table 7:** Mean *Juniperus sabina* concentration (%) of limonene, terpinen-4-ol, citronellic acid methyl ester, and methyl eugenol in the essential oil fractions obtained from the seven distillation timeframes (DT).

DT (min)	Limonene (%)	Terpinen-4-ol (%)	Citronellic Acid Methyl Ester (%)	Methyl Eugenol (%)
3	2.37 a ^1^	0.65 c	2.36 c	0.68 b
5	1.58 ab	4.05 ab	11.00 a	4.87 ab
10	1.97 ab	5.03 a	8.25 ab	6.08 ab
20	1.77 ab	5.36 a	6.23 bc	6.97 a
40	1.49 b	5.54 a	5.24 bc	9.69 a
80	1.54 b	4.65 ab	4.31 c	10.32 a
Control	2.06 ab	2.71 bc	3.85 c	4.70 ab

^1^ Within each column, means followed by the same letter are not significantly different at the 5% level of significance.

**Table 8:** Chemical families of *Juniperus sabina* essential oil (EO) constituents as a function of their elution during hydrodistillation timeframes.

DT (min)	0–3	3–5	5–10	10–20	20–40	40–80	Control 0–80
MH%	90.5	45.7	51.2	48.0	38.6	30.8	69.5
PCH%	2.40	4.60	5.90	6.29	6.36	5.35	4.1
Ph	0.68	4.87	6.08	6.97	10.0	10.3	4.7

DT—Distillation time; MH—Monoterpene hydrocarbons; PCH—polycyclic aromatic hydrocarbons; Ph—phenylpropene.

**Table 9:** Mean oxygen radical absorbance capacity (ORAC) value (uM Trolox Equiv/per g oil) in *Juniperus virginiana*, *J. excelsa*, and *J. sabina* obtained from different distillation times (DT).

DT (min)	*J. virginiana* ORAC	DT (min)	*J. excelsa* ORAC	DT (min)	*J. sabina* ORAC
5	214 b ^1^	5	68.2 b	3	52.8 ab
10	329 a	80	10.3 c	10	55.9 a
40	227 b	160	6.4 cd	20	22.3 c
160	184 b	240	3.4 d	40	35.8 abc
240	205 b	Control	104.1 a	80	29.7 bc
Control	193 b	-	-	Control	28.2 bc

^1^ Within each species, ORAC means followed by the same letter are not significantly different at the 5% level of significance.

## Supplementary Materials

Supplementary File 1
