# Supplementary material for: Hydrodistillation Extraction Kinetics Regression Models for Essential Oil Yield and Composition in Juniperus virginiana, J. excelsa, and J. sabina
Source: Molecules. 2019 Mar 11;24(5):986. doi: 10.3390/molecules24050986 (PMC6429388; doi:10.3390/molecules24050986)
Supplement: Supplementary File 1 [file molecules-24-00986-s001.pdf]

Supplemental material for:

Hydrodistillation extraction kinetics regression models for essential oil yield and composition in *Juniperus virginiana*, *J. excelsa*, and *J. sabina*

Ivanka B. Semerdjieva [1], Santosh Shiwakoti [2], Charles L. Cantrell [3], Valtcho D. Zheljazkov [2,*], Tess Astatkie [4], Vicki Schlegel [5], Tzenka Radoukova [6]

[1] Department of Botany and Agrometeorology, Agricultural University, Mendeleev 12, 4000 Plovdiv, Bulgaria; v_semerdjieva@abv.bg

[2] Crop and Soil Science Department, Oregon State University, 3050 SW Campus Way, 109 Crop Science Building, Corvallis, OR 97331, USA; santoshcwakoti@gmail.com (S.H.); Valtcho.jeliazkov@oregonstate.edu (V.D.Z.)

[3] National Center for Natural Products Research, Agricultural Research Service, United States Department of Agriculture, University, MS 38677, USA; clcantr1@olemiss.edu

[4] Faculty of Agriculture, Dalhousie University, PO Box 550, Truro, NS B2N 5E3, Canada; astatkie@dal.ca

[5] Department of Food Science and Technology, University of Nebraska – Lincoln, 327 Food Technology Complex, Lincoln, NE 68583, USA; vschlegel3@unl.edu

[6] Department of Botany and Methods of Biology Teaching, Faculty of Biology, University of Plovdiv "Paisii Hilendarski", 24 Tzar Assen, 4000 Plovdiv, Bulgaria; kiprei@abv.bg

* Correspondence: Valtcho.jeliazkov@oregonstate.edu

Supplemental Table 1. Observed and reported retention index values for compounds identified by Kovat analysis only.

Compound	Observed RI	Calculated RI	Identification Methods
pregeijerene B	KI = 1276	RI = 1277.56	RI, Kovat
α-thujene	KI = 930	RI = 928.52	RI, Kovat, NIST
δ-cadinene	KI = 1523	RI = 1523.61	RI, Kovat, NIST
elemol	KI = 1550	RI = 1554.91	RI, Kovat, NIST
elemicin	KI = 1557	RI = 1553.35	RI, Kovat, NIST

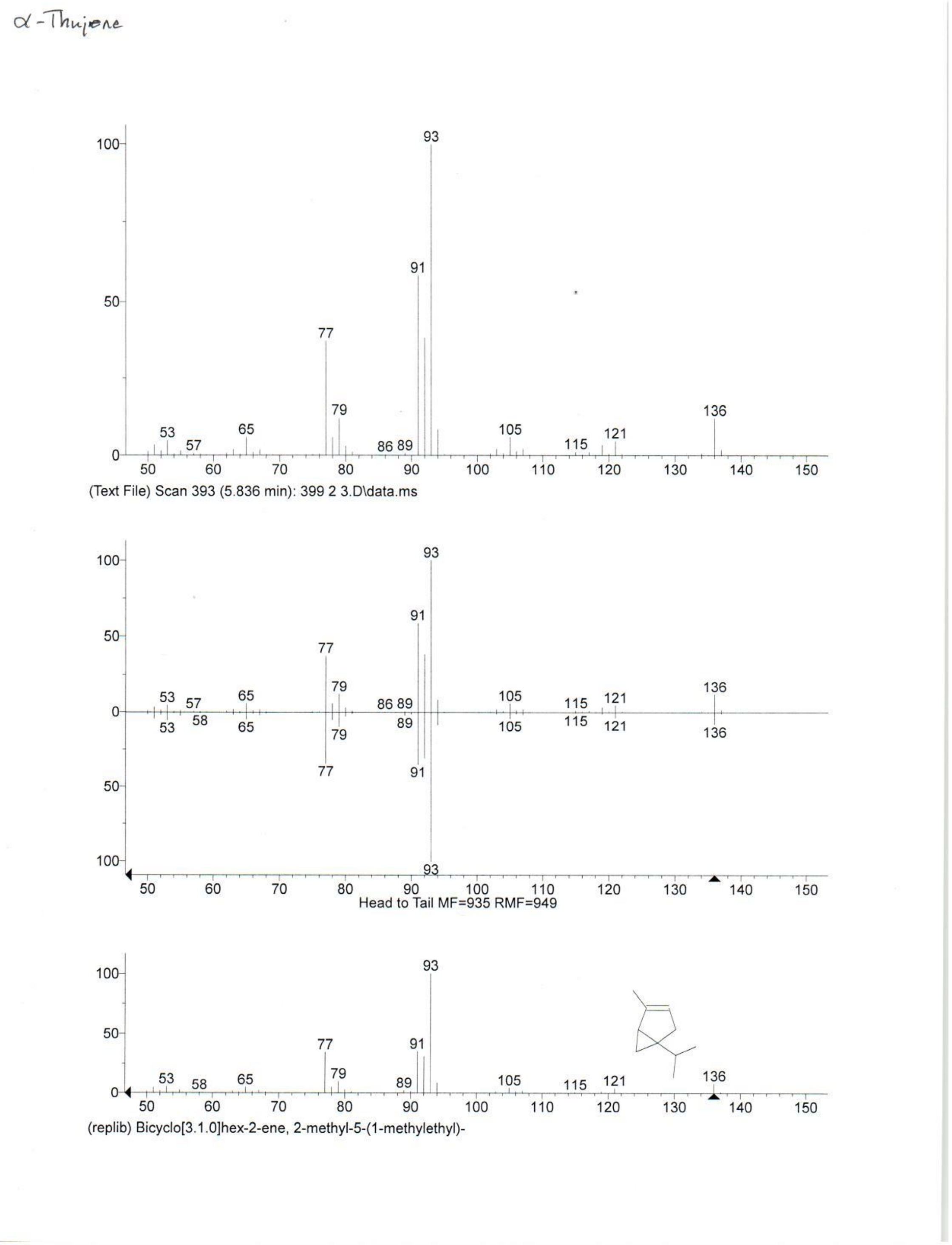

Supplemental Figure 1. Comparison of actual mass spectrum for α-thujene and NIST database mass spectrum.

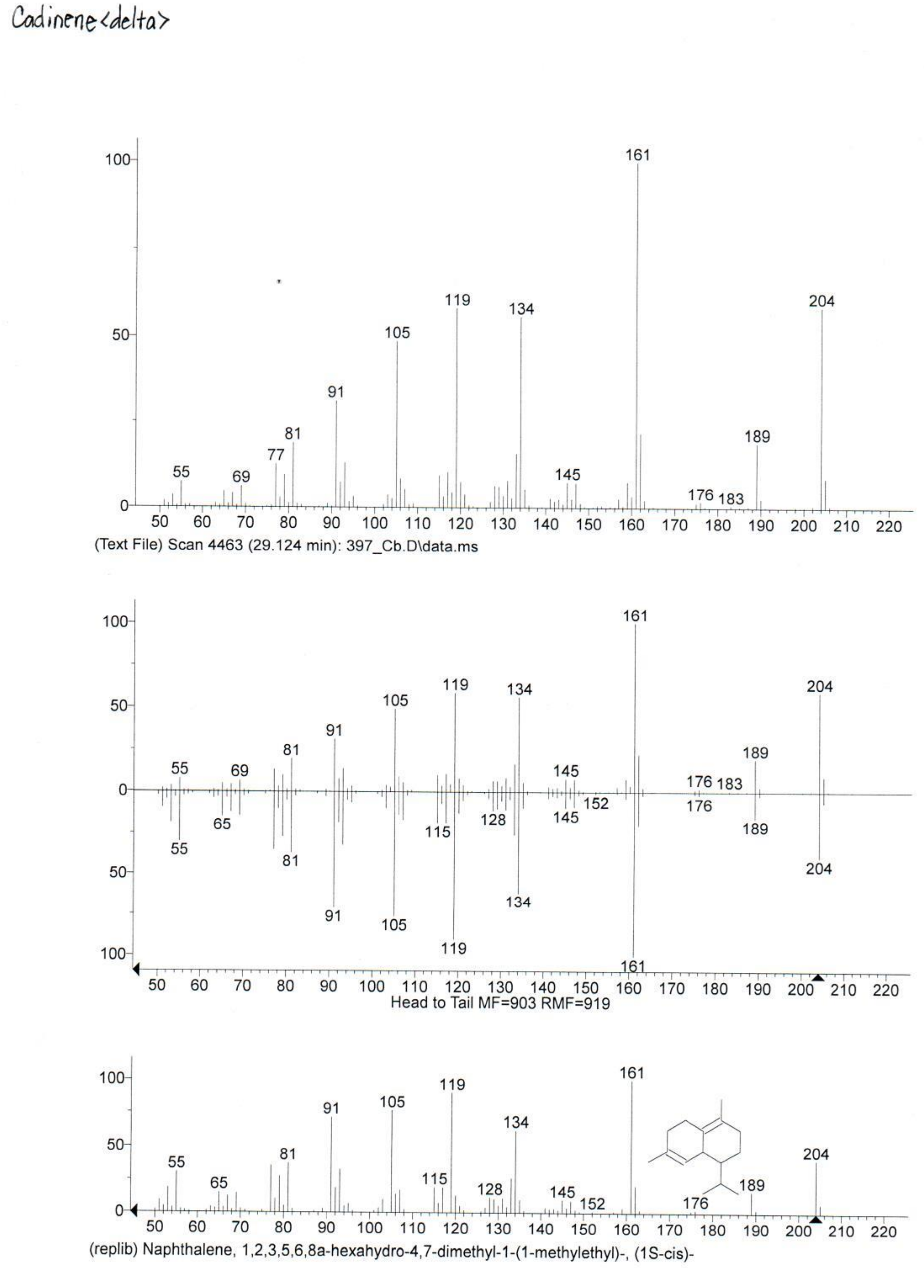

Supplemental Figure 2. Comparison of actual mass spectrum for δ-cadinene and NIST database mass spectrum.

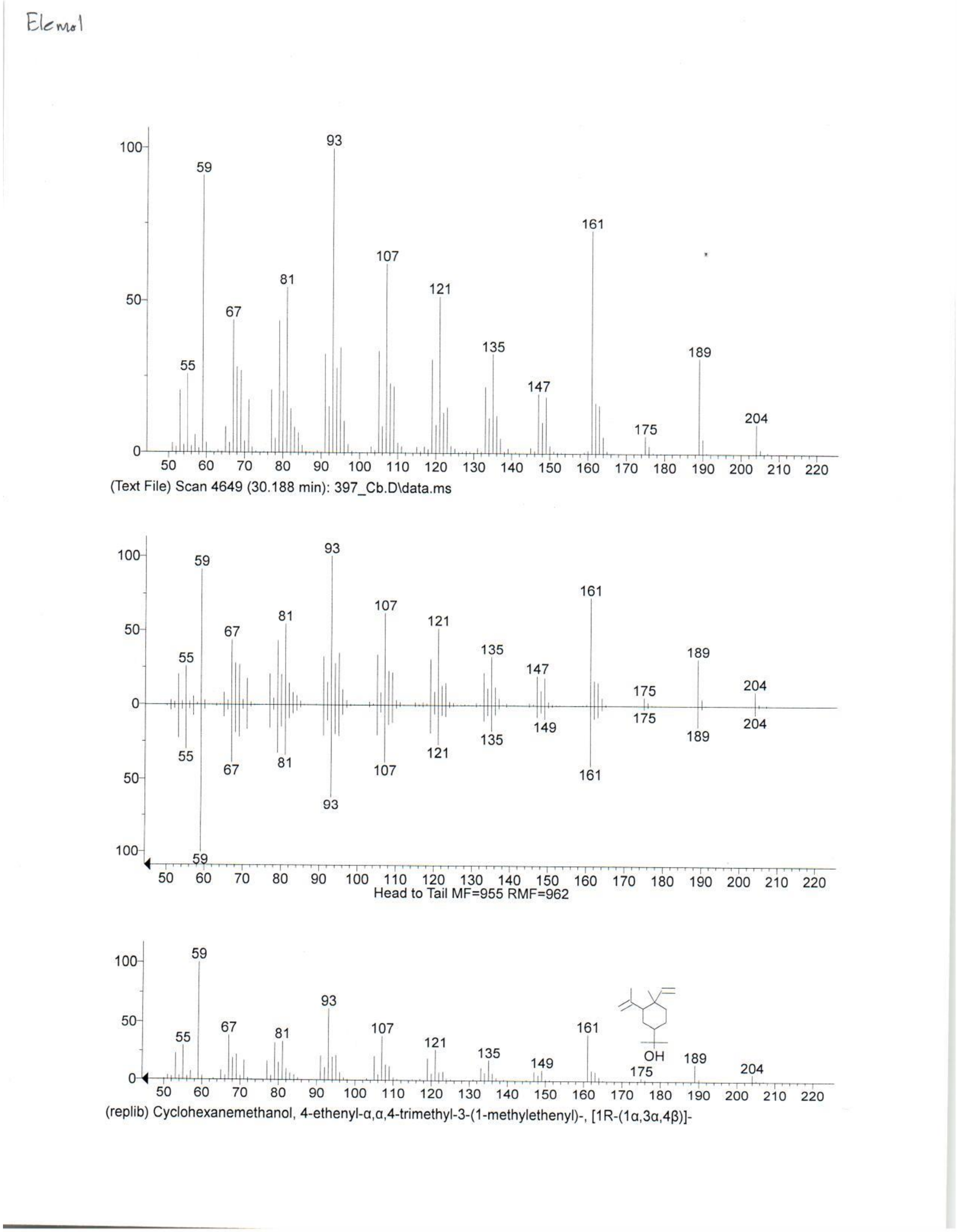

Supplemental Figure 3. Comparison of actual mass spectrum for elemol and NIST database mass spectrum.

Elemicin

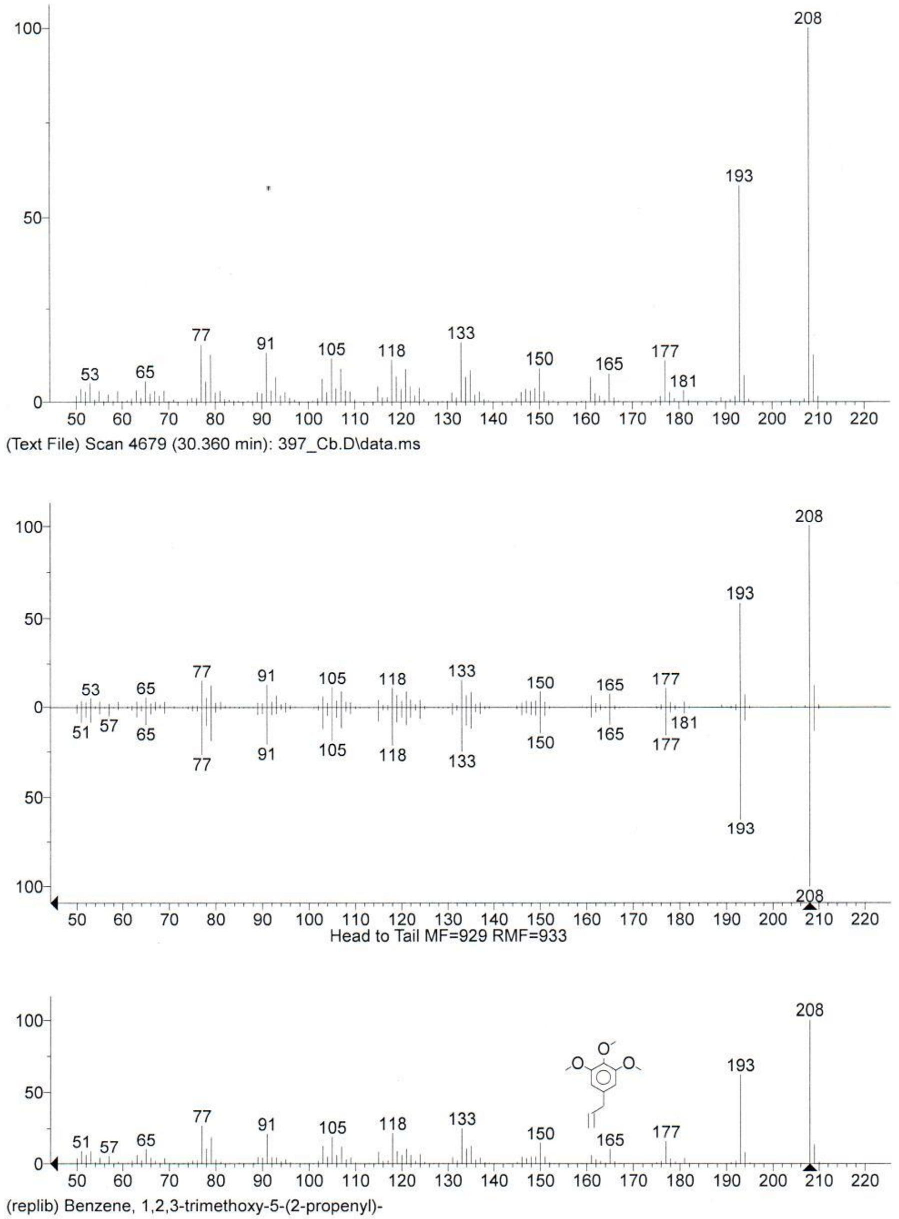

Supplemental Figure 4. Comparison of actual mass spectrum for elemicin and NIST database mass spectrum.

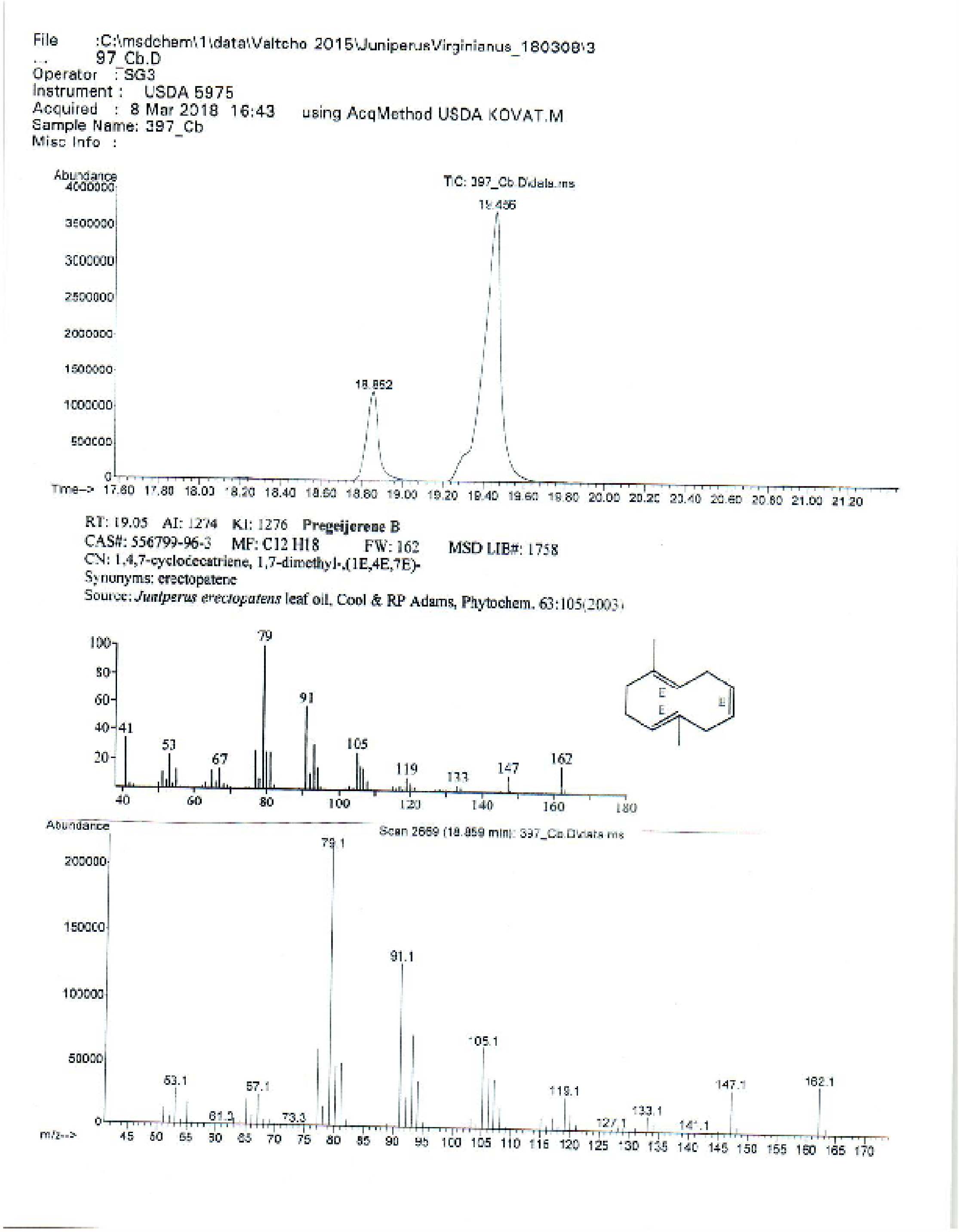

Supplemental Figure 5. Comparison of actual mass spectrum for pregeijerene B and Kovat reported mass spectrum.

Distillation study with Juniperus virginiana from Bulgaria conducted on Jan 4, 2018

Sample #	Timeframe, repliate	Species	g oil
	0-5 min rep 1	*Juniperus virginiana*	0.1195
	0-5min rep 2	*Juniperus virginiana*	0.1056
	0-5min rep 3	*Juniperus virginiana*	0.1330
	5-10min rep1	*Juniperus virginiana*	0.0156
	5-10min rep2	*Juniperus virginiana*	0.0490
	5-10 min rep 3	*Juniperus virginiana*	0.1528
	10-20 min rep1	*Juniperus virginiana*	0.0943
	10-20 min rep2	*Juniperus virginiana*	0.0771
	10-20 min rep 3	*Juniperus virginiana*	0.0280
	20-40 min rep 1	*Juniperus virginiana*	0.1340
	20-40 min rep 2	*Juniperus virginiana*	0.1523
	20-40 min rep 3	*Juniperus virginiana*	0.1809
	40-80 min rep 1	*Juniperus virginiana*	0.1714
	40-80 min rep 2	*Juniperus virginiana*	0.2277
	40-80 min rep 3	*Juniperus virginiana*	0.2130
	80-160 min rep 1	*Juniperus virginiana*	0.1462
	80-160 min rep 2	*Juniperus virginiana*	0.2412
	80-160 min rep 3	*Juniperus virginiana*	0.0675
	160-240 min rep 1	*Juniperus virginiana*	0.1006
	160-240 min rep 2	*Juniperus virginiana*	0.0798
	160-240 min rep 3	*Juniperus virginiana*	0.0586
	Controll 0-240 min	*Juniperus virginiana*	1.1982
	Control 0-240 min	*Juniperus virginiana*	1.0444

Distillation study with Juniperus excelsa from Bulgaria, conducted on Jan 5 2018

Timeframe, replicate	Species	g oil
0-5 min rep 1	*Juniperus excelsa*	0.3552
0-5min rep 2	*Juniperus excelsa*	0.2522
0-5min rep 3	*Juniperus excelsa*	0.2717
5-10min rep1	*Juniperus excelsa*	0.0683
5-10min rep2	*Juniperus excelsa*	0.0665
5-10 min rep 3	*Juniperus excelsa*	0.0747
10-20 min rep1	*Juniperus excelsa*	0.0996
10-20 min rep2	*Juniperus excelsa*	0.0709
10-20 min rep 3	*Juniperus excelsa*	0.0728
20-40 min rep 1	*Juniperus excelsa*	0.0149
20-40 min rep 2	*Juniperus excelsa*	0.0992
20-40 min rep 3	*Juniperus excelsa*	0.1249
40-80 min rep 1	*Juniperus excelsa*	0.0000
40-80 min rep 2	*Juniperus excelsa*	0.0000
40-80 min rep 3	*Juniperus excelsa*	0.0000
80-160 min rep 1	*Juniperus excelsa*	0.0000
80-160 min rep 2	*Juniperus excelsa*	0.0000
80-160 min rep 3	*Juniperus excelsa*	0.0318
160-240 min rep 1	*Juniperus excelsa*	0.0163

160-240 min rep 2	*Juniperus excelsa*	0.0000
160-240 min rep 3	*Juniperus excelsa*	0.0000
Controll 0-240 min	*Juniperus excelsa*	0.7180
Control 0-240 min	*Juniperus excelsa*	1.1434

Distillation study with Juniperus sabina collected in Bulgaria

Replicate, timeframe in mi	Species	g of oil
**rep1-time1; 0-3min**	*Junipers sabina*	0.4625
**rep2-time1; 0-3min**	*Junipers sabina*	0.7038
**rep3-time1; 0-3min**	*Junipers sabina*	0.5532
**rep1-time2; 3-5min**	*Junipers sabina*	0.3036
**rep2-time2; 3-5min**	*Junipers sabina*	0.1110
**rep3-time2; 3-5min**	*Junipers sabina*	0.1753
**rep1-time3; 5-10min**	*Junipers sabina*	0.1710
**rep2-time3; 5-10min**	*Junipers sabina*	0.2388
**rep3-time3; 5-10min**	*Junipers sabina*	0.1878
**rep1-time4; 10-20min**	*Junipers sabina*	0.1089
**rep2-time4; 10-20min**	*Junipers sabina*	0.1188
**rep3-time4; 10-20min**	*Junipers sabina*	0.1125
**rep1-time5; 20-40min**	*Junipers sabina*	0.1430
**rep2-time5; 20-40min**	*Junipers sabina*	0.1887
**rep3-time5; 20-40min**	*Junipers sabina*	0.1033
**rep1-time6; 40-80min**	*Junipers sabina*	0.2273
**rep2-time6; 40-80min**	*Junipers sabina*	0.2248
**rep3-time6; 40-80min**	*Junipers sabina*	0.2236
**Control; 0-80min**	*Junipers sabina*	1.5801
**Control; 0-80min**	*Junipers sabina*	1.7085
